# Chordoma Genetic Aberrations and Targeted Therapies Panorama: A Systematic Literature Review

**DOI:** 10.3390/jcm13092711

**Published:** 2024-05-05

**Authors:** Edoardo Agosti, Sara Antonietti, Marco Zeppieri, Tamara Ius, Alessandro Fiorindi, Alessandro Tel, Massimo Robiony, Pier Paolo Panciani, Marco Maria Fontanella

**Affiliations:** 1 Division of Neurosurgery, Department of Medical and Surgical Specialties, Radiological Sciences and Public Health, University of Brescia, Piazza Spedali Civili 1, 25123 Brescia, Italy; edoardo_agosti@libero.it (E.A.);; 2 Department of Ophthalmology, University Hospital of Udine, p.le S. Maria della Misericordia 15, 33100 Udine, Italy; 3 Neurosurgery Unit, Head-Neck and NeuroScience Department, University Hospital of Udine, p.le S. Maria della Misericordia 15, 33100 Udine, Italy; 4 Clinic of Maxillofacial Surgery, Head-Neck and NeuroScience Department, University Hospital of Udine, p.le S. Maria della Misericordia 15, 33100 Udine, Italy

**Keywords:** chordoma, genetic and molecular aberrations, systematic reviews, treatment implications

## Abstract

**Background**: Chordomas pose a challenge in treatment due to their local invasiveness, high recurrence, and potential lethality. Despite being slow-growing and rarely metastasizing, these tumors often resist conventional chemotherapies (CTs) and radiotherapies (RTs), making surgical resection a crucial intervention. However, achieving radical resection for chordomas is seldom possible, presenting therapeutic challenges. The accurate diagnosis of these tumors is vital for their distinct prognoses, yet differentiation is hindered by overlapping radiological and histopathological features. Fortunately, recent molecular and genetic studies, including extracranial location analysis, offer valuable insights for precise diagnosis. This literature review delves into the genetic aberrations and molecular biology of chordomas, aiming to provide an overview of more successful therapeutic strategies. **Methods**: A systematic search was conducted across major medical databases (PubMed, Embase, and Cochrane Library) up to 28 January 2023. The search strategy utilized relevant Medical Subject Heading (MeSH) terms and keywords related to “chordomas”, “molecular biology”, “gene aberrations”, and “target therapies”. The studies included in this review consist of preclinical cell studies, case reports, case series, randomized controlled trials, non-randomized controlled trials, and cohort studies reporting on genetic and biological aberrations in chordomas. **Results**: Of the initial 297 articles identified, 40 articles were included in the article. Two tables highlighted clinical studies and ongoing clinical trials, encompassing 18 and 22 studies, respectively. The clinical studies involved 185 patients diagnosed with chordomas. The tumor sites were predominantly sacral (*n* = 8, 44.4%), followed by clivus (*n* = 7, 38.9%) and lumbar spine (*n* = 3, 16.7%). Primary treatments preceding targeted therapies included surgery (*n* = 10, 55.6%), RT (*n* = 9, 50.0%), and systemic treatments (*n* = 7, 38.9%). Various agents targeting specific molecular pathways were analyzed in the studies, such as imatinib (a tyrosine kinase inhibitor), erlotinib, and bevacizumab, which target EGFR/VEGFR. Common adverse events included fatigue (47.1%), skin reactions (32.4%), hypertension (23.5%), diarrhea (17.6%), and thyroid abnormalities (5.9%). Clinical outcomes were systematically assessed based on progression-free survival (PFS), overall survival (OS), and tumor response evaluated using RECIST or CHOI criteria. Notably, stable disease (SD) occurred in 58.1% of cases, and partial responses (PRs) were observed in 28.2% of patients, while 13.7% experienced disease progression (PD) despite targeted therapy. Among the 22 clinical trials included in the analysis, Phase II trials were the most prevalent (40.9%), followed by I-II trials (31.8%) and Phase I trials (27.3%). PD-1 inhibitors were the most frequently utilized, appearing in 50% of the trials, followed by PD-L1 inhibitors (36.4%), CTLA-4 inhibitors (22.7%), and mTOR inhibitors (13.6%). **Conclusions**: This systematic review provides an extensive overview of the state of targeted therapy for chordomas, highlighting their potential to stabilize the illness and enhance clinical outcomes.

## 1. Introduction

Chordomas are rare, slow-growing tumors that arise from remnants of the notochord, typically occurring along the axial skeleton, with the sacrum being the most common location [1]. Despite their infrequency, chordomas pose significant challenges in clinical management due to their locally aggressive nature and propensity for recurrence. The epidemiology of chordomas reflects their rarity, with an estimated annual incidence of 0.08 per 100,000 individuals [2]. These tumors predominantly affect adults, with a peak incidence observed in the fifth and sixth decades of life [3].

The clinical management of chordomas is particularly daunting, marked by the tumors’ resistance to conventional therapeutic modalities, including surgery, radiation therapy, and CT [4]. Surgical resection, the primary treatment option, is often limited by the intricate anatomical locations of chordomas and the need to achieve radical excision while preserving neurological function. Furthermore, despite advancements in RT techniques, chordomas exhibit resistance to radiation, contributing to a high recurrence rate [5]. Chemotherapeutic agents have demonstrated limited efficacy, emphasizing the urgent need for alternative therapeutic strategies. The prognosis for chordoma patients remains bleak, with a five-year survival rate ranging from 50% to 70%. This poor prognosis is exacerbated by the challenges in achieving complete tumor resection and the lack of effective systemic therapies. The critical issue lies in the intrinsic resistance of chordomas to conventional treatments, necessitating a paradigm shift in therapeutic approaches [6].

Recent studies have shed light on potential molecular targets in chordomas, offering a glimmer of hope for the development of effective targeted therapies. Understanding the molecular underpinnings of chordomas is crucial, as it provides insights into the mechanisms of resistance and identifies vulnerabilities that can be exploited for therapeutic purposes [7]. The emergence of targeted therapies represents a promising avenue for addressing the unmet clinical needs in chordoma treatment. Several studies have explored the molecular landscape of chordomas, unraveling intricate signaling pathways and aberrant molecular processes that contribute to their pathogenesis. These investigations have identified potential molecular targets, ranging from cell surface receptors to intracellular signaling cascades. Despite this progress, the lack of a standardized and universally accepted treatment protocol underscores the complexity of treating chordomas [8].

Recognizing the urgent requirement to overcome the high resistance of chordomas to current therapies, there is a growing body of literature investigating factors contributing to resistance and exploring novel treatment strategies. These strategies predominantly involve targeted therapies that aim to disrupt specific molecular pathways implicated in chordoma progression [9]. Initial reports in the literature have highlighted various targeted treatments showing promise in preclinical and clinical settings. These treatments often focus on inhibiting specific molecules or pathways that play a pivotal role in chordoma development and progression. However, the absence of a consensus on the optimal treatment approach reflects the intricate nature of chordoma biology and the need for a comprehensive evaluation of existing evidence [10]. Moreover, an updated systematic review of the main targeted treatments available for chordoma treatment is still needed.

In light of the evolving landscape of targeted therapies for chordomas, this systematic literature review aims to provide a comprehensive synthesis of current knowledge. By reviewing and synthesizing the available evidence on the molecular mechanisms underlying targeted therapies in chordomas, we aim to identify trends, commonalities, and gaps in the literature.

## 2. Materials and Methods

### 2.1. Literature Review

The systematic review was performed following the Preferred Reporting Items for Systematic Reviews and Meta-Analysis (PRISMA) guidelines [11]. The study protocol was not registered. Two authors (E.A. and S.A.) performed a systematically comprehensive literature search of the databases PubMed, Ovid MEDLINE, and Scopus. The first literature search was performed on 5 January 2024, and the search was updated on 19 February 2024. A combination of keyword searches was performed to generate a search strategy. The search keywords, including “chordomas”, “targeted therapies”, “outcomes”, and “adverse events”, utilized both AND and OR combinations. Retrieval of studies employed MeSH terms and Boolean operators: (chordomas) AND (targeted therapies OR targeted treatments) AND (outcomes OR survival OR adverse events). Additional relevant articles were identified through scrutinizing the references of the selected papers. Inclusion criteria for study selection encompassed the following: (1) studies written in the English language; (2) clinical studies on targeted therapies for chordomas; and (3) studies providing insights into clinical outcomes and/or adverse events. Conversely, exclusion criteria included the following: (1) editorials, literature reviews, and meta-analyses; and (2) studies lacking clear delineation of methods and/or results.

The inventory of identified studies was integrated into Endnote X9, where duplicate entries were expunged. The results were meticulously scrutinized independently by two researchers (E.A. and S.A.) adhering to the predefined inclusion and exclusion criteria. Any disparities were arbitrated by a third reviewer (P.P.P.). Subsequently, articles meeting the eligibility criteria underwent a thorough examination during the full-text screening process.

### 2.2. Data Extraction

Each study’s details were systematically extracted, encompassing the following information: authorship, publication year, patient cohort size, previous therapeutic interventions, targeted molecular entity, studied agent, and clinical outcomes (i.e., PFS, OS, tumor response evaluated with RECIST and/or CHOI criteria, and reported adverse events).

### 2.3. Outcomes

The primary outcomes focused on characterizing the main targeted treatments (including target, agent, dosage, and duration of treatment). Secondary outcomes encompassed clinical outcomes.

### 2.4. Risk-of-Bias Assessment

**Figure 1:**
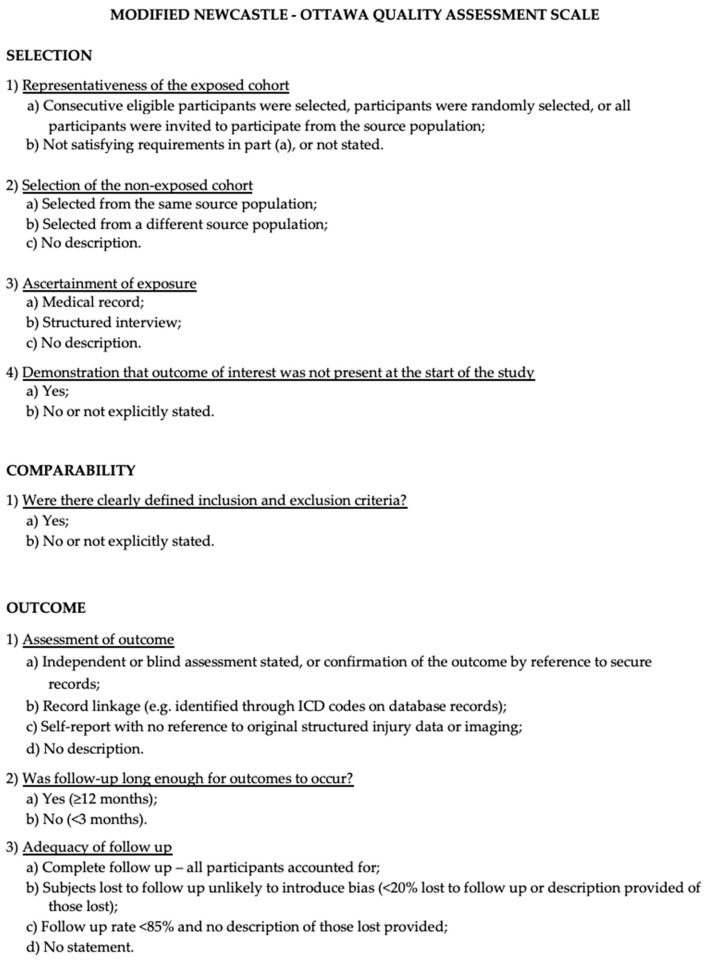
The modified NOS.

The Newcastle–Ottawa Scale (NOS) was used to assess the quality of the included studies [12]. Quality assessment was performed by assessing the selection criteria, comparability of the study, and outcome assessment. The ideal score was 9. Higher scores indicated better quality of studies. Studies receiving 7 or more points were considered high-quality studies. Two authors (E.A. and P.P.P.) performed the quality assessment independently. When discrepancies arose, papers were re-examined by the third author (Figure 1).

### 2.5. Statistical Analysis

Descriptive statistics were reported, including ranges and percentages. All statistical analyses were performed using the R statistical package v3.4.1 (http://www.r-project.org (accessed on 22 February 2024).

## 3. Results

### 3.1. Literature Review

**Figure 2:**
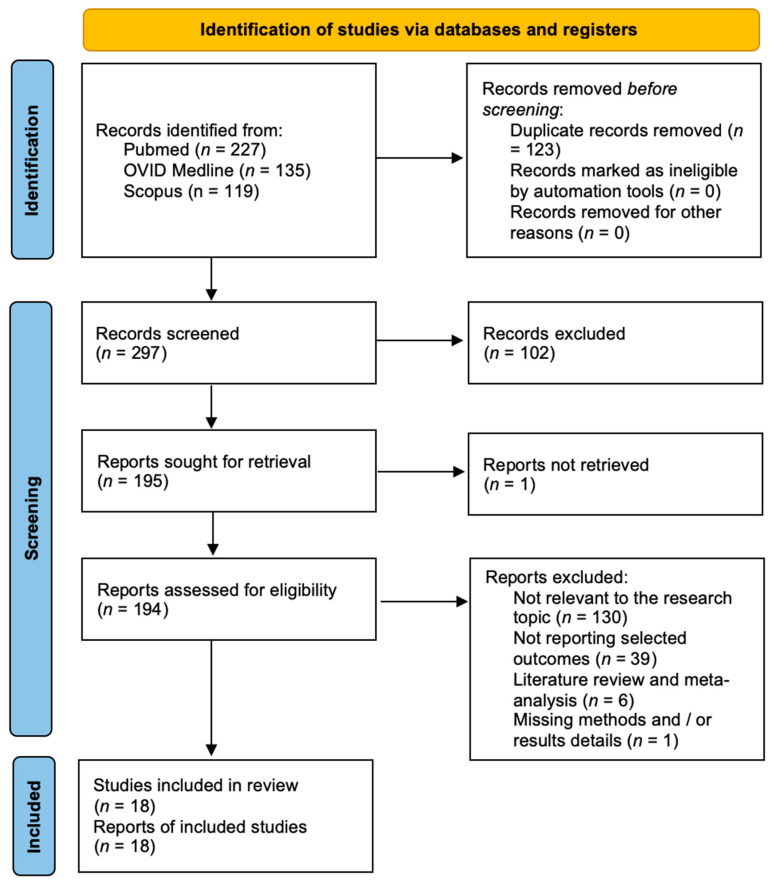
PRISMA flowchart.

A total of 297 papers were identified after duplicate removal. After title and abstract analysis, 195 articles were identified for full-text analysis. Eligibility was assessed for 194 articles and ascertained for 40 articles. The remaining 154 articles were excluded for the following reasons: (1) not relevant to the research topic (110 articles), (2) articles not reporting selected outcomes (37 articles), (3) systematic literature review or meta-analysis (5 articles), and (4) lack of method and/or results details (1 article). All studies included in the analysis had at least one or more outcome measures available for one or more of the patient groups analyzed. Figure 2 shows the flowchart according to the PRISMA statement.

**Figure A1:**
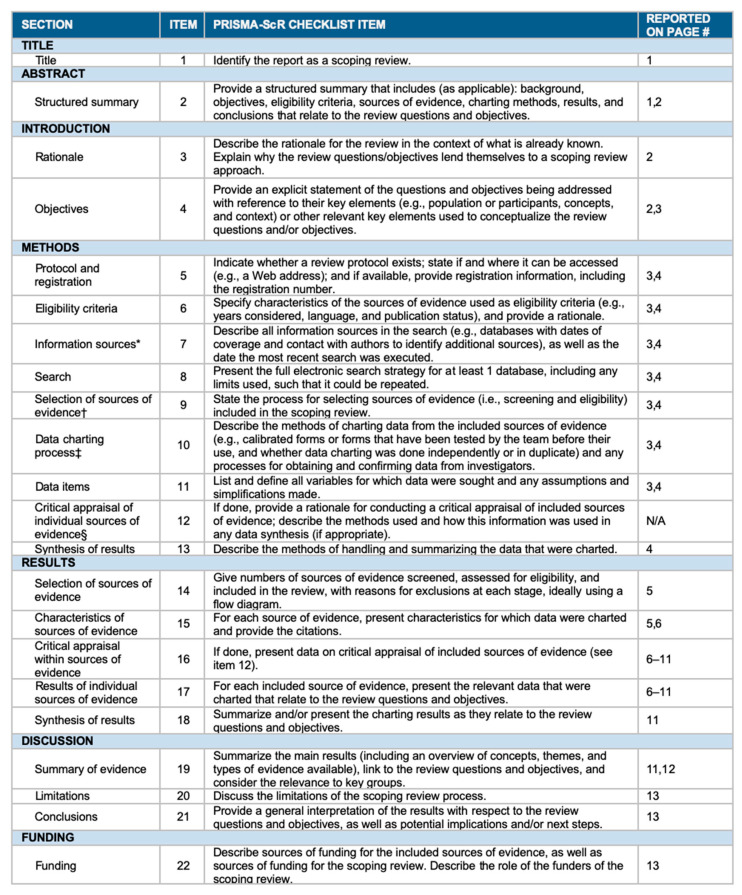
The PRISMA-ScR checklist. * Where sources of evidence (see second footnote) are compiled from, such as bibliographic databases, social media platforms, and websites. † A more inclusive/heterogeneous term used to account for the different types of evidence or data sources (e.g., quantitative and/or qualitative research, expert opinion, and policy documents) that may be eligible in a scoping review as opposed to only studies. This is not to be confused with information sources (see first footnote). ‡ The frameworks by Arksey and O’Malley (6) and Levac and colleagues (7) and the JBI guidance (4, 5) refer to the process of data extraction in a scoping review as data charting. § The process of systematically examining research evidence to assess its validity, results, and relevance before using it to inform a decision. This term is used for items 12 and 19 instead of “risk of bias” (which is more applicable to systematic reviews of interventions) to include and acknowledge the various sources of evidence that may be used in a scoping review (e.g., quantitative and/or qualitative research, expert opinion, and policy document).

The PRISMA Extension for Scoping Reviews (PRISMA-ScR) checklist is available in Appendix A, Figure A1.

### 3.2. Data Analysis

A summary of the included studies reporting on targeted therapies for chordomas is presented in Table 1 for clinical studies.

The comprehensive systematic review encompassed data from 18 clinical studies conducted between 2013 and 2023, collectively involving 185 patients diagnosed with chordomas. Patient distribution across these studies reflected the rarity of the condition and the limited research available. The tumor sites were predominantly sacral (*n* = 8, 44.4%), followed by clivus (*n* = 7, 38.9%) and lumbar spine (*n* = 3, 16.7%). Primary treatments preceding targeted therapies included surgery (*n* = 10, 55.6%), RT (*n* = 9, 50.0%), and systemic treatments (*n* = 7, 38.9%). Various agents targeting specific molecular pathways implicated in chordoma progression and resistance were employed in the studies. Imatinib, a tyrosine kinase inhibitor, emerged as the most frequently used agent, administered at a dosage of 1500 mg daily. Other agents included sorafenib, targeting VEGFR, PDGFR, c-kit, and RET, and erlotinib and bevacizumab, targeting EGFR/VEGFR. These agents were prescribed at dosages such as 400 mg orally twice daily and 150 mg daily, respectively. The duration of targeted therapy exhibited considerable variability across studies, with median durations ranging from 4 to 13 months. Adverse events associated with targeted therapies were reported across multiple studies. Common adverse events included fatigue (47.1%), skin reactions (rash, hand–foot syndrome) (32.4%), hypertension (23.5%), diarrhea (17.6%), and thyroid abnormalities (5.9%). Clinical outcomes were systematically assessed based on PFS, OS, and tumor response evaluated using RECIST or CHOI criteria. Notably, SD was the predominant response to targeted therapies across the studies, occurring in 58.1% of cases. PR was observed in 28.2% of patients, while 13.7% experienced PD despite targeted therapy. Imatinib, as the most extensively studied agent, demonstrated promising efficacy in terms of SD and PR. Sorafenib also exhibited notable disease control in chordoma patients, with 63.0% experiencing SD. Immunotherapies targeting PD-1, PD-L1, and CTLA-4, such as pembrolizumab, nivolumab, and tremelimumab, demonstrated variable responses. Some patients achieved PR or SD, constituting 12.5% and 68.8% of cases, respectively.

A summary of the included studies reporting on targeted therapies for chordomas is presented in Table 2 for ongoing clinical trials.

Among the 22 clinical trials included in the analysis, the majority were initiated in recent years, with 2017 being the most prolific year, featuring in 27.3% of the studies. This temporal concentration suggests a contemporary emphasis on advancing therapeutic strategies against chordomas. Phase II trials were the most prevalent (40.9%), followed by I-II trials (31.8%) and Phase I trials (27.3%). This distribution indicates a substantial focus on investigating the efficacy of targeted therapies in more advanced stages, reflecting a progressive evolution in chordoma research. Analyzing the agent classes employed in these trials, the data demonstrated a diverse range of targeted approaches. PD-1 inhibitors were the most frequently utilized, appearing in 50% of the trials, followed by PD-L1 inhibitors (36.4%), CTLA-4 inhibitors (22.7%), and mTOR inhibitors (13.6%). These percentages highlight a predominant interest in immunotherapy, particularly in modulating the PD-1 pathway, as a leading avenue for chordoma treatment. The exploration of CTLA-4 and mTOR inhibitors also underlines the diverse strategies being pursued to address the complexity of chordoma pathogenesis. Examining the specific agents used in the trials, nivolumab emerged as the most frequently employed, featured in 36.4% of the studies. This indicates a significant focus on PD-1 inhibition, with nivolumab being a key player in chordoma-targeted therapies. Other agents such as ipilimumab, durvalumab, and tremelimumab were also recurrent, showcasing the exploration of combination therapies and the concurrent targeting of multiple pathways. Delving into the therapeutic targets, the data revealed PD-1 as the most commonly addressed, targeted in 45.5% of the trials. Other notable targets included CTLA-4 (22.7%), mTOR (18.2%), and IDH (4.5%). These percentages underscore the emphasis on modulating immune checkpoint pathways, as well as targeting key signaling and metabolic pathways implicated in chordoma development.

## 4. Discussion

### 4.1. Immunotherapy for Chordomas

The clinical care of chordomas presents unique challenges due to the tumors’ resistance to standard therapeutic approaches, such as RT, surgery, and CT scanning. A series of target therapies have been added to the previous one. Our study has identified a number of therapeutic strategies that can improve clinical outcomes, including immunotherapy.

#### 4.1.1. Immune Checkpoint Inhibitors

Immunotherapy has emerged as a promising frontier in the quest for effective chordoma treatments, with a particular focus on immune checkpoint inhibitors [27,31]. The International Immunocancer Registry (ICIR) data, meticulously detailed by Ramos-Casals et al. [21], not only underscores the potential of immune checkpoint inhibitors but also sheds light on the intricate immunological responses. In their exploration, the study highlights instances of sicca/Sjögren’s syndrome triggered by PD-1/PD-L1 checkpoint inhibitors, providing crucial insights into the dynamic interplay between immunomodulation and treatment responses in chordomas [21]. Building on this foundation, the study delves into the immunologic correlates of the abscopal effect in chordomas following EZH2 inhibition and RT. This emphasis on the abscopal effect, where localized radiation triggers a systemic antitumor immune response, signifies the multifaceted nature of immunomodulation in chordoma treatment [25]. The integration of immunotherapy with traditional modalities such as RT represents a synergistic approach that holds promise for improved therapeutic outcomes [32].

The pioneering work of Migliorini et al. [19]. further strengthens the case for immunotherapy in challenging chordoma scenarios. Their investigation into clinical responses in relapsing chordoma cases, post the failure of standard therapies, underscores the potential of immunomodulatory interventions. The findings advocate for the consideration of immunotherapeutic strategies as salvage options for refractory chordomas, opening new avenues for exploration and clinical application. Moreover, the correlation between clinical responses to nivolumab and immunogenic recognition of brachyury in INI1-deficient pediatric chordomas, as articulated by Williamson et al. [23], provides valuable insights into the immunogenomic landscape of chordomas. Brachyury, as an embryonic transcription factor, has been implicated in pathogenesis [24]. Understanding its role in immunogenic recognition offers a targeted approach that aligns immunotherapy with the specific molecular characteristics of chordomas [13,14,15,16,17,18,20,33].

In terms of clinical efficacy, PD-1/PD-L1 inhibitors are between 10% and 30% effective in common solid tumors [34]. On the other hand, immunotherapy is quite expensive and may postpone the illness if other treatments are ineffective.

Thus, one of the main areas of focus for immunotherapy research is enhancing the effectiveness of ICIs to enable precision anticancer therapy [35].

According to the study of Tan et al. [35], the detection of therapeutic response and prognostic biomarkers, a combination of two or more ICs, the definition of a Performance Forecasting Model for JVs, and the conversion of Tregs in inflammatory cells are valid strategies to improve the effectiveness of immunotherapy. Diarrhea, itching, and fatigue are listed as the main adverse effects of PD-1 inhibitors.

The intricate connection between immune responses and chordoma outcomes is further underscored by studies exploring the immune microenvironment [36]. Feng et al. [37] reported on the expression of PD-L1 and the prevalence of tumor-infiltrating lymphocytes (TILs) in chordomas, providing additional layers of information crucial for refining immunotherapeutic strategies. The presence and activity of TILs have been associated with improved outcomes in various cancers [38].

#### 4.1.2. CAR-T Cell Therapy

In the realm of innovative therapeutic approaches, chimeric antigen receptor T-cell (CAR-T) therapy stands out as a transformative strategy. The groundbreaking work of Beard et al. [39]. showcases the successful targeting of CSPG4 in diverse cancer histologies, exemplifying the potential of CAR-T cell therapy in addressing the inherent heterogeneity of chordomas. This approach moves beyond the conventional ‘one-size-fits-all’ paradigm, offering a tailored solution that aligns with the unique molecular landscape of individual chordoma cases [39].

Expanding on this frontier, Long et al. [40]. contribute significantly by exploring B7-H3 as a target for CAR-T cell therapy in skull base chordoma. This innovative endeavor demonstrates the adaptability of CAR-T therapy to different molecular targets, presenting an evolving repertoire of precision treatments. B7-H3, as a novel target, expands the scope of CAR-T therapy, potentially addressing cases where traditional modalities fall short. This adaptability positions CAR-T therapy as a dynamic and responsive approach in the ever-evolving landscape of chordoma treatment [40].

CAR-T cells have completely changed how some cancers are treated. CAR-T cell function depends on the choice of antigen [41]. The CAR-T cells’ selective pressure causes tumor cells to downregulate antigens. On-target off-tumor consequences can happen even with proper antigen targeting, leading to related toxicity. It is difficult to make CAR-T cells travel to and infiltrate solid tumors. The immunosuppressive microenvironment of cancers may exacerbate this challenge [41].

In comparison to other cancer treatments, the results from follow-up trials that are currently available indicate a comparatively low risk of developing secondary malignancies after receiving CAR-T cell therapy [42].

Because of meticulous safety monitoring during the trial phase, acute immunological adverse events associated with CAR-T cell therapy, such as cytokine release syndrome and immune effector cell-associated neurotoxicity syndrome, are well documented. This has enabled the development of novel treatments for these complications and modifications to the CAR-T cell therapy to mitigate their deleterious effects [42].

The integration of CAR-T cell therapy into the chordoma treatment paradigm reflects a paradigm shift towards personalized and targeted interventions. By leveraging the specificity of CAR-T cells, these approaches hold the promise of enhanced efficacy with reduced off-target effects. As the field continues to explore novel antigens and optimize CAR-T cell engineering, the potential for further innovations in chordoma therapy becomes increasingly apparent [43,44].

### 4.2. Genomic Insights Informing Targeted Approaches

The systematic literature review meticulously dissects pivotal genomic insights, laying the groundwork for nuanced and targeted therapeutic approaches in chordomas. Forrest et al. [45] identified INI1 deficiency in pediatric cancers, offering a comprehensive understanding of the genomic and immunologic landscape of INI1-deficient chordomas. This revelation is paramount for tailoring targeted interventions to the unique molecular profiles of individual patients, ushering in an era of precision medicine for chordoma therapy [46]. The study by Forrest et al. [45] delves into the intricate genomic and immunologic characterization of INI1-deficient pediatric cancers, unraveling the complexities of this specific subset of chordomas. This information not only refines our comprehension of chordoma pathogenesis but also unveils potential vulnerabilities that can be exploited for therapeutic purposes. Understanding the genomic underpinnings becomes the cornerstone for developing targeted strategies, aligning interventions with the underlying molecular intricacies driving chordoma development and progression [46,47].

Expanding on the genomic landscape, the roles of the embryonic transcription factor BRACHYURY in tumorigenesis and progression, as articulated by Chen et al. [48], present additional dimensions for targeted interventions. BRACHYURY, known for its involvement in chordoma pathogenesis, becomes a focal point for therapeutic exploration. The study provides insights into the molecular machinery driving chordoma development, offering a rational basis for the design of targeted therapies aimed at disrupting key tumorigenic pathways [48,49,50]. Furthermore, Schoenfeld et al. [51] contribute to the repertoire of potential targets by highlighting the prognostic significance of chondroitin sulfate proteoglycan 4 (CSPG4), an emerging biomarker in chordomas. The study not only underscores the clinical relevance of CSPG4 but also positions it as a potential candidate for targeted therapies. Incorporating biomarkers such as CSPG4 into the diagnostic and therapeutic landscape allows for a more refined and personalized approach to chordoma treatment, where interventions are tailored based on the specific genetic makeup of individual tumors [51].

By classifying cancer molecularly according to changes in the genome and transcriptome, new biomarkers for diagnosis, prognosis, and therapy response may become apparent [52].

It will take ongoing interdisciplinary cooperation between oncologists, pathologists, fundamental scientists, and computational biologists to translate the cancer genome and transcriptome for patients. To support the continued tumor sequencing in the clinic, data-sharing networks to enable precision cancer care in the clinic, and profiling efforts for fundamental genomics research, more funds and resources are required. An integrated network will be needed for clinical trials for precision cancer medicine in order to manage tumor samples and clinical data and provide access to new treatments [52].

The dynamic evolution of genomic profiling in chordomas is instrumental in identifying novel therapeutic targets. The rich interplay of genetic alterations and signaling pathways uncovered by these studies provides a roadmap for the development of innovative and targeted interventions. The promise lies not only in treating chordomas more effectively but also in mitigating potential side effects by precisely targeting cancer cells while sparing healthy tissues [22,24,26,27,28,29,30,47,53,54,55,56].

### 4.3. CDK4/6 Treatment

Rana et al. [57] listed the CDK inhibitors undergoing clinical studies at the moment. The potency and selectivity of proteolysis targeting chimeras (PROTACs) are more determined by the linker length, composition, and choice of E3 ligand than kinase inhibitors, whose effectiveness is directly correlated with binding affinity. However, creating a powerful and targeted degrader requires considerable optimization, and designing any PROTAC is an iterative process. Phase II clinical trials are underway for the PROTACs ARV-471 and ARV-110, while Phase I clinical trials are also underway for the PROTACs KT-474 and NX-2127. The scientific community is given the impetus to keep exploring PROTACs as a different approach to treatment by these early clinical candidates. Future research will concentrate on creating disease-specific PROTACs by the use of ligands that target tissue-specific E3 ligases or by investigating novel disease-specific proteins of interest.

### 4.4. Location-Based Molecular Target and Potential Specific Treatments

According to the study of Salle et al. [58], the molecular distinctions between sacral chordomas and skull base chordomas have very seldom been examined in the research. Jager et al. [59] examined the expression of HOX genes, a gene family involved in the development of the anterior–posterior body axis, in cell cultures generated from chordomas of both locations. Compared to clival tumors, the scientists noted that SCs expressed more of the genes HOXA7, HOXA9, and HOXA10. These findings might suggest a connection between this anatomical location and a gene control mechanism. In this way, Salle et al.’s [58] research demonstrated a correlation between the chordoma’s location (skull base or sacrum) and the kind of genetic abnormalities (increase or loss of CNV).

### 4.5. Limitations

Our scoping review is limited by the heterogeneity of study designs of the included works, their retrospective nature, the fragmentary data reported, and their relatively small samples. Despite the promising clinical results of targeted therapies, the study showed that more research and standardization in the management of chordomas are necessary due to the variation in treatment regimens, patient groups, and reporting standards among studies.

## 5. Conclusions

This systematic review offers a comprehensive overview of the current landscape of targeted therapies for chordomas, demonstrating their potential for disease stabilization and improved clinical outcomes. The nuanced details of treatment regimens, patient responses, and adverse events emphasize the need for ongoing research efforts to refine therapeutic approaches and establish standardized guidelines for chordoma management. The analysis revealed that despite the encouraging clinical outcomes observed with targeted therapies, the heterogeneity in treatment regimens, patient populations, and reporting standards across studies underscored the need for further research and standardization in the management of chordomas. Additionally, the long-term efficacy and safety of targeted therapies warrant continued investigation, particularly in the context of combination therapies and personalized treatment approaches. Collaborative efforts across the scientific and medical community will be crucial for advancing our understanding and treatment of chordomas in the era of precision medicine.

## Figures and Tables

**Table 1:** Summary of clinical studies included in the systematic literature review reporting on targeted therapies for chordomas.

Author, Year	Patients N	Age (Mean)	Female (N, %)	Tumor Site	Precedent Treatment (N)		Target Therapy				Outcomes			
						Drug	Target	Dose	Median Duration (Months)	AEs	Choi’s	Recist	mPFS (Months)	mOS (Months)
Stacchiotti et al. [13] 2013	18	61 (median)	8, 44.4%	Sacrum (12 pts), clivus (2 pts), lumbar and cervical spine (4 pts)	Imatinib	Lapatinib	Tyrosine kinase	1500 mg QD	5	Anemia (2 pts), fatigue (2 pts), rash (5 pts), hypertension (2 pts), and thromboembolism (1 pt)	PR (6 pts), SD (7 pts), PD (5 pts)	SD (15 pts), PD (3 pts)	8	25
Asklund et al. [14] 2014	3	51	2, 66.7%	Sacrum (1 pt), clivus (2 pts)	Surgery, RT	Erlotinib/bevacizumab	EGFR/ VEGFR	150 mg QD/10 mg/kg once weekly	13	Fatigue	N/A	SD (2 pts)	N/A	N/A
Bompas et al. [15] 2015	27	64 (median)	10, 37.0%	Sacrum (20 pts), lumbar spine (3 pts), clivus (3 pts), dorsal spine (1 pt)	Surgery (18 pts), RT (18 pts), systemic treatments (12 pts)	Sorafenib	VEGFR, PDGFR, c-kit, RET	400 mg orally BID	9	Hand–foot syndrome (5 pts), other skin reactions (2 pts), mucositis (2 pts), fatigue (4 pts), loss of appetite (1 pt), weight loss (4 pts), diarrhea (5 pts), arterial hypertension (6 pts), thyrotoxicosis (1 pt), lymphopenia (3 pts), hypokalemia (1 pt)	N/A	PR (7 pts), SD (5 pts), PD (1 pt)	73% at 12 months	86.5% at 12 months
Aleksic et al. [16] 2016	1	65	1, 100%	Lumbar spine	Surgery, RT	Linsitinib/erlotinib	IGF-1R/EGFR	50 mg QD → BID /100 mg QD	61	Dry skin, abnormal hair and eyelash growth, bilateral hallux paronychia, diarrhea	N/A	PD	N/A	N/A
Lebellec et al. [17] 2017	80	59.0 (median)	34, 42.5%	Sacrum (50 pts), lumbar and cervical spine (12 pts), clivus (18 pts)	Surgery (58 pts), RT, CT (11 pts)	Imatinib (62 pts), sorafenib (11 pts), erlotinib (5 pts), sunitinib (1 pt), temsirolimus (1 pt)	Tyrosine kinases	N/A	7	N/A	N/A	SD (58 pts), PD (10 pts), PR (5 pts)	9	4
Jägersberg et al. [18] 2017	3	56	2, 46%	Clivus	Surgery, RT	Imatinib (2 pts), pazopanib/nivolumab (1 pt)	Tyrosine kinases, PD-1	N/A	N/A	Pneumopathy	N/A	N/A	27	N/A
Migliorini et al. [19] 2017	3	54	2, 66.7%	Clivus (2 pts), cervical spinal (1 pt)	Surgery, RT, CT, everolimus, erlotinib/cetuximab, pazopanib	Pembrolizumab (1 pt), MVX-ONCO-1 (1 pt), nivolumab	PD-1, GM-CSFR	200 mg, 3 mg/Kg	7	Vitiligo	N/A	N/A	9	N/A
Bilusic et al. [20] 2019	5	59.7	N/A	N/A	N/A	HuMax-IL8	IL8	4/8/16/32 mg/kg IV every 2 weeks	6	N/A	N/A	SD (4 pts), PD (1 pts)	N/A	N/A
Ramos-Casals et al. [21] 2019	1	51	1, 100%	N/A	N/A	Durvalumab	PD-L1	N/A	4	Sicca/Sjögren’s syndrome	N/A	N/A	N/A	N/A
Wu et al. [22] 2020	1	52	0, 0%	Lumbosacral spine	Surgery	Pembrolizumab	PD-1	200 mg every 3 weeks	10	Abnormal liver function, hyperglycemia	N/A	PR	N/A	N/A
Williamson et al. [23] 2021	1	N/A	N/A	Clivus, C1	CT	Nivolumab	PD-1	N/A	N/A	N/A	N/A	PR	N/A	N/A
Chen et al. [24] 2021	1	77	0, 0%	Sacrum	Surgery, RT	Sintilimab, anlotinib	PD-1, VEGFR, FGFR, PDGFR c-kit	N/A	N/A	Myocarditis	N/A	N/A	N/A	<1
Gounder et al. [25] 2022	1	25	1, 100%	Sacrum	Surgery, RT, tazemetostat	Nivolumab/ipililumab	PD-1/CTLA-4	N/A	N/A	N/A	N/A	N/A	>4	N/A
Somaiah et al. [26] 2022	5	N/A	2, 46%	N/A	N/A	Tramelimumab/durvalumab	CTLA-4/PD-L1	75 mg IV/1500 mg	N/A	Colitis, pneumonitis, abdominal pain, myocarditis	N/A	CR (1 pt), SD (3 pts), PD (1 pt)	14	N/A
Bishop et al. [27] 2022	17	56.0 (median)	4, 24.0%	N/A	N/A	Pembrolizumab (9 pts), durvalumab/tremelimumab (5 pts), nivolumab/IL-2 (1 pt), FAZ053 (2 pts)	PD-1, PD-L1, CTLA-4	200 mg IV every 3 weeks, 150 mg/75 mg IV every 4 weeks	8	Dermatologic (2 pts), endocrine (2 pts), sicca syndrome-related (2 pts), myocarditis and myositis (1 pt), colitis (1 pt), pneumonitis (1 pt)	N/A	PD (2 pts), SD (11 pts), PR (3 pts), CR (1 pt)	56.0% at 12 months	87.0% at 12 months
Ibodeng et al. [28] 2022	1	77	0, 0%	Clivus	Surgery, RT	Pembrolizumab/imatinib	PD-1/tyrosine kinase	200 mg every 3 weeks/400 mg QD	N/A	Hypotension, severe fatigue, dyspnea	N/A	N/A	N/A	N/A
Kesari et al. [29] 2023	1	66	0, 0%	Sacrum	CT, nivolumab	AdAPT-001/pembrolizumab	TGFβ/PD-1	2.5 × 10^11^ vp/200 mg IV every 3 weeks	13	N/A	N/A	PD	N/A	N/A
Blay et al. [30] 2023	34	N/A	23, 45.0%	N/A	N/A	Pembrolizumab	PD-1	200 mg IV	24 (maximum)	N/A	N/A	PR (4 pts), PD (6 pts), SD (35 pts)	7	N/A

Abbreviations: BID = twice daily; CT = chemotherapy; EGFR = epidermal growth factor receptor; IGF-1R= type 1 insulin-like growth factor receptor; IV = intravenous; N/A = not applicable; PD = progressive disease; PR = partial response; pt = patient; QD = once daily; RT = radiotherapy; SD = stable disease; VEGFR = vascular endothelial growth factor; vp = viral particles.

**Table 2:** Summary of ongoing clinical trials included in the systematic literature review reporting on targeted therapies for chordomas.

NCT Number	Year	Phase	Agent Class	Agent	Target
NCT01267955	2010	II	Hh pathway inhibitor	Vismodegib	Hh pathway
NCT02193503	2014	I	Personalized cancer vaccine	MVX-ONCO-1	APCs
NCT02601950	2015	II	EZH2 inhibitor	Tazemetostat	EZH2
NCT02383498	2015	II	Cancer vaccine	GI-6301 Vaccine (yeast—brachyury)	APCs
NCT02936102	2016	I	PD-L1 inhibitor, PD-1 inhibitor	FAZ053 PDR001	PD-L1, PD-1
NCT02746081	2016	I	IDH inhibitor	BAY1436032	IDH
NCT02815995	2016	II	PD-L1 inhibitor, CTLA-4 inhibitor	Durvalumab, tremelimumab	PD-L1 CTLA-4
NCT03173950	2017	II	PD-1 inhibitor	Nivolumab	PD-1
NCT02989636	2017		PD-1 inhibitor	Nivolumab	PD-1
NCT03190174	2017	I-II	mTOR inhibitor, PD-1 inhibitor	Nab-rapamycin, nivolumab	mTOR, PD-1
NCT02834013	2017	II	CTLA-4 PD-1 inhibitor	Ipilimumab, nivolumab	PD-1, CTLA-4
NCT02982486	2017	II	PD-1 inhibitor, CTLA-4 inhibitor	Nivolumab, ipilimumab	PD-1, CTLA-4
NCT03190174	2017	I-II	PD-1 inhibitor, mTOR inhibitor	Nivolumab, ABI-009	PD-1, mTOR
NCT02989636	2017	I	PD-1 inhibitor	Nivolumab	PD-1
NCT03647423	2018	I-II	Cancer vaccine	NANT chordoma vaccine	APCs
NCT03595228	2018	II	Transgenic vaccine	BN-brachyury	APCs
NCT03623854	2019	II	PD-1 inhibitor, LAG-3 inhibitor	Nivolumab, relatlimab	PD-1, LAG-3
NCT03886311	2019	II	PD-1 inhibitor	Talimogene laherparepvec, nivolumab, and trabectedin	PD-1, minor groove of DNA
NCT04246671	2020	I	Cancer vaccine HER2 inhibitor	TAEK-VAC-HerBy vaccine, HER2 antibodies	HER2/neu
NCT04278781	2020	II	IDH1 inhibitor	AG-120	IDH1
NCT04416568	2020	II	CTLA-4 PD-1 inhibitor	Ipilimumab, nivolumab	CTLA-4, PD-1
NCT05286801	2022	I-II	PD-L1 inhibitor Anti-TIGIT monoclonal antibody	Atezolizumab, tiragolumab	PD-L1, TIGIT

Abbreviations: APCs = antigen-presenting cells; CTLA-4 = Cytotoxic T-Lymphocyte Antigen 4; EZH2 = enhancer of zeste homolog 2; IDH1/2 = isocitrate dehydrogenase 1/2; LAG = lymphocyte- activation gene 3; PD-1 = programmed cell death protein 1; PD-L1 = programmed cell death ligand 1; TIGIT = T cell immunoreceptor with immunoglobulin and ITIM domain.

## Data Availability

Data are available in a publicly accessible repository.
